# Cardiac Metastasis from Myxoid Liposarcoma Managed Successfully with Chemotherapy and Radiotherapy: Case Report and Review of the Literature

**DOI:** 10.3390/curroncol31090398

**Published:** 2024-09-12

**Authors:** Georgios M. Stergiopoulos, Brittany L. Siontis, Ivy A. Petersen, Matthew T. Houdek, Thanh P. Ho, Scott H. Okuno, Steven I. Robinson

**Affiliations:** 1 Department of Molecular Medicine, Mayo Clinic, Rochester, MN 55905, USA; stergiopoulos.georgios@mayo.edu; 2 Department of Medical Oncology, Mayo Clinic, Rochester, MN 55905, USA; 3 Department of Radiation Oncology, Mayo Clinic, Rochester, MN 55905, USA; 4 Department of Orthopedic Surgery, Mayo Clinic, Rochester, MN 55905, USA

**Keywords:** cardiac metastasis, chemotherapy, radiotherapy, liposarcoma, myxoid liposarcoma, pericardial metastasis, sarcoma

## Abstract

Background: Liposarcoma, one of the most prevalent sarcoma histologies, is recognized for its tendency for extra-pulmonary metastases. While oligometastatic cardiac disease is rarely reported, it poses a unique challenge as oligometastatic sarcomas are often managed with surgical resection. Case Report: We present a case of a 62-year-old man diagnosed with an oligometastatic myxoid liposarcoma (MLPS) to the heart 19 years after the primary tumor resection from the lower limb. The metastatic mass, situated in the pericardium adjacent and infiltrating the left ventricle, was not managed surgically but with a combination of chemotherapy and radiotherapy. The patient’s disease remains stable to date, for more than 10 years. Literature Review: We conducted a review of the literature to determine the preferred management approach for solitary cardiac metastases of sarcomas. We also conducted an in-depth analysis focusing on reported cases of MLPS metastasizing to the heart, aiming to extract pertinent data regarding the patient characteristics and the corresponding management strategies. Conclusions: Although clinical diagnoses of solitary or oligometastatic cardiac metastases from sarcomas are infrequent, this case underscores the significance of aggressive management employing chemotherapy and radiotherapy for chemosensitive and radiosensitive sarcomas, especially when surgical removal is high-risk. Furthermore, it challenges the notion that surgery is the exclusive therapeutic option leading to long-term clinical benefit in patients with recurrent sarcomas.

## 1. Background

Soft tissue sarcomas are a heterogeneous group of malignancies featuring approximately 100 distinct histological subtypes [1]. Liposarcoma is the most common soft tissue sarcoma, making up to 17–25% of all soft tissue sarcomas [2,3]. Malignant adipocyte tumors can be differentiated according to the most recent World Health Organization (WHO) classification in well-differentiated liposarcoma (lipoma-like, sclerosing, inflammatory), dedifferentiated liposarcoma, myxoid liposarcoma (MLPS), pleomorphic liposarcoma, and myxoid pleomorphic liposarcoma [4]. Myxoid (and round cell) is the second most common type of liposarcoma, comprising about 35% of all liposarcoma cases. The median age at diagnosis is 45 years old, and the most common primary locations are in the lower extremities and buttocks [5].

The cytogenetics of MLPS have been well characterized. Specifically, this tumor is driven by the translocation of DDIT3 (DNA damage-inducible transcript 3, also known as C/EBP-homologous protein/CHOP). Most cases (~90%) are driven by the (12;16) (q13;p11) chromosomal translocation that results in the DDIT3-FUS (fused in sarcoma, also known as translocated in sarcoma [TLS]) gene fusion. The remaining cases present with a (12;22) (q13;q12) that leads to a DDIT3-EWSR1 (Ewing sarcoma breakpoint region 1) gene fusion, with EWSR1 being closely related to FUS [6,7].

MLPS is metastatic in 14–33% of the cases [8,9,10,11], but unlike other sarcomas, it has a great propensity for extra-pulmonary metastases [8,9,10,11]. Common sites of metastasis include soft tissue and bones, lungs, abdominal solid organs (e.g., liver, or less frequently, pancreas and kidneys), and lymph nodes [8,9,10,11]. Cardiac metastases are extremely rare with only 46 cases being reported worldwide [12,13,14,15,16,17,18,19,20,21,22,23,24,25,26,27,28,29,30,31,32,33,34,35,36,37,38,39,40,41,42,43,44,45,46,47,48,49,50,51,52,53,54,55] (Table 1).

In general, sarcoma metastasis to the heart is considered to be a rare phenomenon as most of them remain asymptomatic and go unrecognized; nonetheless, their incidence in post-mortem analysis might be up to 25% [56]. Once diagnosed, cardiac metastases warrant urgent treatment, given the high mortality rate [57,58]. However, complete surgical resection poses significant risks due to the difficult anatomic location, resulting in high perioperative mortality rates and uncertain benefits for patients [59]. Individual tumor characteristics, location of the metastasis, risk of imminent death (e.g., secondary to arrhythmia, tamponade, thromboembolic events, heart failure), risk of perioperative mortality, and patients’ wishes should guide the therapeutic decision-making process. 

In this paper, we present the case of a patient with solitary cardiac metastasis 19 years after the initial diagnosis and management of an MLPS of the thigh, who has been in long-term remission (>10 years) following aggressive chemotherapy and radiotherapy to the cardiac oligometastatic deposit, without any surgical intervention. To our knowledge, this patient, apart from being the longest survivor following cardiac metastasis of MLPS, is the only reported case with an oligometastatic cardiac liposarcoma metastasis managed without surgery (other than two cases where the patients died soon after the diagnosis and before the therapeutic plan was initiated [16,18]) (In the case described by Motevalli et al., the patient was initially diagnosed with a mediastinal metastasis via sonography. The metastasis was confirmed to be cardiac in the post-mortem autopsy).

## 2. Case Report

We report the case of a 62-year-old man with MLPS of the lower limb with a late oligometastatic tumor to the heart. The metastasis was managed with chemotherapy and radiotherapy, resulting in stable disease to this day, with more than 10 years of follow-up.

This patient was diagnosed at the age of 43 with primary MLPS confined to the vastus lateralis muscle of the thigh. Tumor resection was followed by adjuvant radiotherapy, and he remained disease-free for 19 years. Post-treatment, the patient had left lower extremity lymphedema and neuropathy but no local relapse.

**Figure 1:**
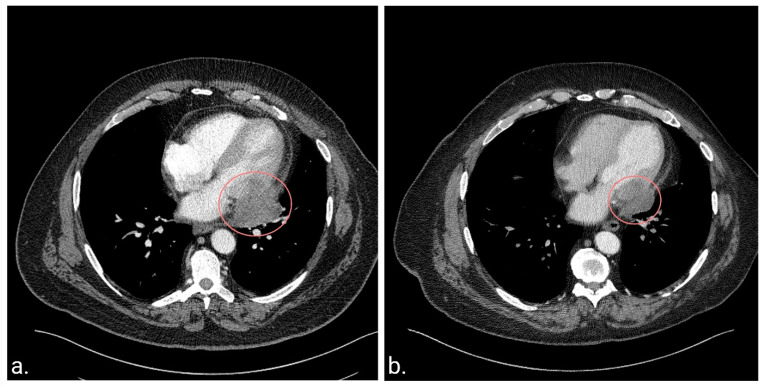
Chest computerized tomography (CT) scans at diagnosis of the cardiac metastasis and 10 years post-treatment. (**a**). CT scan before the initiation of chemotherapy and radiotherapy demonstrating a heterogeneous mass located in the pericardium and extending into the left ventricle. (**b**). The latest CT scan 10.5 years after diagnosis demonstrating a residual soft tissue mass centered in the left posterolateral pericardium, which has remained stable since the completion of therapy.

At the age of 62 years, while evaluating his lymphedema symptoms, abdominal and pelvic computed tomography scans (CT) revealed a pericardial mass. The patient did not experience any symptoms attributed to the cardiac mass at the time of diagnosis. Subsequent chest CT (Figure 1a) and cardiac magnetic resonance imaging (MRI) revealed a solitary 5.2 × 4.7 × 3.5 cm mass involving the pericardium and infiltrating the lateral wall of the left ventricle. Cardiac MRI was selected to complement the CT findings due to its superiority in characterizing soft tissue and its high specificity in distinguishing between benign pseudomasses and malignant cardiac tumors [60,61]. Additionally, MRI becomes particularly relevant when recurrent soft-tissue sarcoma is suspected due to its high diagnostic accuracy [62]. 

**Figure 2:**
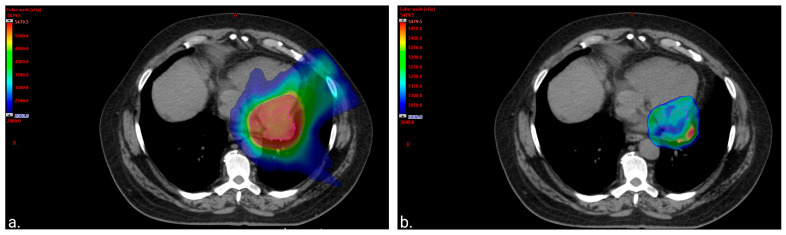
Color wash images to visually represent the distribution of the radiation. (**a**). Color wash image of 20 gray (Gy) volume displaying sparing of the lung and anterior heart. The volume of the total lung receiving at least 20 Gy (total lung V20Gy) was equal to 10.6% (left lung V20Gy = 23.4% and right lung V20Gy = 0%). The heart mean dose was equal to 20 Gy with only 75 cubic centimeters (cc) of the treatment volume overlapping with the heart and 150 cc of the heart getting ≥30 Gy. (**b**). Color wash image of 50 Gy volume displaying sparing of the apex of the left ventricle from the prescription dose.

Metastatic MLPS was confirmed following a video-assisted thoracoscopic surgery (VATS). At the VATS procedure, it was noted that the tumor was not attached to the pericardium at the site of biopsy. The treatment plan included neo-adjuvant chemo- and radiotherapy prior to determining the feasibility of surgical resection, as the original tumor extent was not felt to be amenable to local excision. The patient received four cycles of doxorubicin (30 mg/m^2^) and ifosfamide (3750 mg/m^2^) on days 1 and 2 (every 21–28 days) with mesna (750 mg/m^2^) and growth factor support. This resulted in a modest (~20%) decrease in maximal tumor dimension. He subsequently received 50 Gray (Gy) in 25 fractions, using an intensity-modulated radiotherapy (IMRT) technique, sparing the heart and lungs of high doses (Figure 2). This was delivered with two concomitant cycles of radiosensitizing chemotherapy with mitomycin (6 mg/m^2^) and cisplatin (45 mg/m^2^) on day 1 (every 28 days), leading to a decrease in tumor size from 5 × 3.7 cm to 4 × 2.5 cm. Doxorubicin was omitted from the radiosensitizing regimen to minimize the expected cardiotoxicity related to chest irradiation, and the patient was closely monitored but did not display any signs of declining cardiac function. In anticipation of surgery, additional chemotherapy with gemcitabine (900 mg/m^2^) on day 1 and gemcitabine (900 mg/m^2^) with docetaxel (75 mg/m^2^) on day 8 (every 21 days) was initiated. Cycle 3 was dose-reduced by 25%, and the patient received steroids for presumed chemotherapy-induced pneumonitis. Following cycle 3, he developed worsening pulmonary toxicity with a differential diagnosis, including infection and worsening inflammation; thus, chemotherapy was discontinued.

CT scan 8 weeks after chemotherapy discontinuation showed a slight increase in tumor size, but subsequent imaging 6 weeks later revealed tumor stability. Surgical resection was deemed high-risk, and as the patient had stable disease with a low likelihood of available chemotherapeutic modalities further reducing the size of the mass, observation was recommended. Surveillance included CT chest, abdomen, and pelvis every 2–3 months for 1.5 years, every 6 months until 5 years, then annually. The patient’s disease has remained stable to date for >10 years since the completion of therapy (Figure 1b). Notably, previous treatment was well-tolerated in terms of cardiac toxicity, with the patient showing no signs or symptoms of declining cardiac function, and a cardiac MRI performed five years post-treatment revealed only mild hypokinesis of the basal lateral left ventricular wall, with a preserved left ventricular ejection fraction of 57%.

## 3. Discussion

Cardiac metastasis from sarcoma is rare; thus, there is no consensus on a treatment approach. 

In our initial literature review, we aimed to assess whether surgical resection is indeed the preferred method for managing solitary cardiac metastases of sarcomas. Employing the terms “Sarcoma(s)” and “Cardiac metastasis/es” on PubMed, we identified 161 published cases (in case reports and case series) of sarcomas metastasizing to the heart. After excluding the cases where the metastases were diagnosed post-mortem (*n* = 6) or at the time of the primary diagnosis (*n* = 36), we analyzed the remaining cases (*n* = 119). Among these, approximately one-third of the patients had isolated cardiac metastases (*n* = 41). Since surgical resection of cardiac metastasis seems to be the anecdotal recommendation in the field [12,63], only a minority of the reviewed cases (*n* = 13, 32.5%) did not undergo surgery. In most of these reports, (*n* = 11, 84.6%) surgical resection was not pursued due to the patient’s status, the patient’s preference, or the inoperable nature of the lesion. Thus, no conclusions can be drawn regarding the efficacy of medical versus surgical management in this patient group.

Cardiac metastases may pose an imminent risk of death, and in such cases, urgent surgical intervention may be warranted [38,48,59,64]. However, in all other instances, surgical resection should not be presented as the exclusive therapeutic modality as it often fails to provide benefit to the patients [59]. Specifically, cases have been described when surgery was attempted, but complete resection was not possible [34,41]. Additionally, even when negative surgical margins had been achieved, local recurrences occurred [20,21,44,65,66,67], in some cases as soon as prior to the initiation of the adjuvant therapy [66]. Ultimately, intracardiac procedures are complex and carry a high risk of intra- or perioperative mortality [35,38,68,69]. Particularly for MLPS, despite the anatomic challenges, surgical resection was attempted in approximately two-thirds of reported cardiac metastases (Table 1). For those who did not undergo surgery, the most common reasons were patient death soon after diagnosis or heavy metastatic burden.

Notably, our literature review revealed cases of patients with cardiac sarcoma metastases treated with radiotherapy, chemotherapy, and/or immunotherapy, achieving stable disease, or even having partial and complete responses without surgical intervention [37,54,70,71,72]. One of these patients had complete radiological resolution on MRI of the cardiac recurrence of a malignant fibrous histiocytoma following treatment with high-dose chemotherapy followed by peripheral blood progenitor cell transplant and immunotherapy with interleukin-2 and 13-cis-retinoic acid [72]. Supporting these data, a single-institution retrospective study in Japan, where none of the patients with sarcoma had resection of cardiac metastasis, suggested that radiotherapy might provide an alternative local treatment option as the median survival of patients receiving radiotherapy was 10.5 months compared to 3.5 months for those who did not [73]. Additionally, the authors claimed that a total dose of more than 45 Gy should be given to achieve the best clinical response [73]. 

Particularly for MLPS, which is among the most chemosensitive [74] and radiosensitive [75] sarcomas, only two cases have been described in which metastatic cardiac MLPS has been treated with radiotherapy, and both responded to treatment [37,54]. In one of them, the patient had multifocal disease and was initially treated with six cycles of doxorubicin (60–75 mg/m^2^). Even though the patient had stable disease, he received adjuvant radiotherapy (35Gy) in 15 fractions to the cardiac lesion due to concerns for arrhythmias with a higher dose. The cardiac lesion remained stable, but new metastatic lesions appeared in the meantime [37]. The other patient, who had multifocal disease and symptoms of heart failure attributed to his cardiac metastasis, had complete resolution of the heart failure symptoms following radiotherapy with 40 Gy [54] over 30 days. Additionally, two cases have been reported in which cardiac MLPS metastases were treated solely with chemotherapy (etoposide or cyclophosphamide, dacarbazine, vincristine, adriamycin) [40,45]. Neither patient responded to treatment, but both had extensive disease at the time of diagnosis, and one of them received single-agent etoposide, which is inferior to standard anthracycline-based chemotherapy [76].

To the best of our knowledge, no other case of solitary cardiac MLPS has been managed primarily with radiotherapy and chemotherapy. The encouraging outcome of our patient, who is by far the longest-reported survivor without disease progression following cardiac metastasis of liposarcoma, is supported by a case described by Pino et al., who reported a patient with a right atrial metastasis of an MLPS which recurred in the atrium one year after surgical management [21]. The recurrence was managed with radiotherapy, leading to complete regression followed by chemotherapy (radiation dose and chemotherapy agents not reported), and at the 6-month follow-up, the patient remained asymptomatic [21].

## 4. Conclusions

To conclude, the decade-long disease stability observed in our patient without surgical intervention remains particularly noteworthy even when accounting for certain positive prognostic factors of this patient. These prognostic factors included a prolonged disease-free interval, a solitary metastasis for which the patient was asymptomatic, and the chemosensitive and radiosensitive tumor type.

Moreover, this case report highlights the importance of multidisciplinary care and underscores that definitive management of oligometastatic sarcoma metastasis, especially when surgical removal is high-risk (e.g., in the heart, brain, or liver hilum), can include chemotherapy and radiotherapy. 

## Figures and Tables

**Table 1:** Published cases of myxoid liposarcoma metastatic to the heart.

Year Published	Authors	Histology (Location) of the Primary Tumor	Cardiac Metastasis Diagnosis Age in Years	Location(s) of Cardiac Metastasis	Time to Cardiac Metastasis	Solitary Cardiac Metastasis/Additional Metastatic Sites	Cardiac Surgery (Provided Rationale When Surgery Was Not Performed)	Outcome Following the Diagnosis of Cardiac Metastasis	Ref.
2024	Stergiopoulos et al.	Myxoid liposarcoma (thigh)	62	LV, pericardium	19 years	Solitary cardiac	No (high-risk surgical procedure)	Patient still alive with stable disease at 11-year follow-up	Present publication
2020	Ikuta et al.	Myxoid liposarcoma (thigh)	40	LV	2 years	Solitary cardiac	Yes	Confirmed death 2 years later	[12]
2020	Bezak et al.	Myxoid liposarcoma (gluteal region)	52	IVS	3 years	Solitary cardiac	Yes	Patient still alive at 3-week follow-up	[13]
2019	Porres-Aguilar et al.	Myxoid liposarcoma (thigh)	63	RV	At initial diagnosis	Thigh (primary site)	No (poor surgical candidacy)	Confirmed death 4 days later	[14]
2018	Passhak et al.	Myxoid liposarcoma (ankle)	49	LV	9 years	Abdomen	Yes	Patient still alive under chemotherapy at 3-year follow-up	[15]
2017	Motevalli et al.	Myxoid liposarcoma (lower limb)	46	LV, pericardium	16 years	Solitary cardiac	No (expired soon after admission)	Confirmed death a few hours later	[16]
2016	Dendramis et al.	Dedifferentiated liposarcoma (pleura)	44	LV	At initial diagnosis	Multiple metastases	No (poor prognosis)	Confirmed death 3 months later	[17]
2015	Farmer et al.	Myxoid liposarcoma (lower limb)	61	LV	17 years	Solitary cardiac	No (tumor deemed as nonresectable)	Confirmed death soon after diagnosis	[18]
2014	Xu et al.	Myxoid liposarcoma (thigh)	60	RV, PA	20 years	Solitary cardiac	Yes	-	[19]
2014	Vajtai et al.	Dedifferentiated liposarcoma (suprarenal retroperitoneal)	57	RA, IVC	4 months	IVC	No (expired soon after diagnosis)	Confirmed death 2 months later	[20]
2013	Pino et al.	Myxoid liposarcoma (shoulder)	36	LUPV, LA, LV	2 years	Solitary cardiac	Yes	Confirmed death 2 months later	[21]
2013	Pino et al.	Myxoid liposarcoma (lung)	35	RA	3 years	Solitary cardiac	Yes	Patient still alive at 1.5-year follow-up	[21]
2013	Pino et al.	Round cell liposarcoma (popliteal fossa)	23	Pericardium	3 years	Solitary cardiac	Yes	Confirmed death 1.5 months later	[21]
2013	Mottahedi et al.	Round cell liposarcoma (knee) (HG)	50	RA, RV, SVC	4 years	Solitary cardiac	Yes	Patient still alive at 1-year follow-up	[22]
2012	Agaimy et al.	Myxoid liposarcoma (thigh)	65	RA, IVC	3 years	Solitary cardiac	Yes	Confirmed death 2 months later	[23]
2012	Fernandez-Golfin et al.	Myxoid liposarcoma (lower limb)	68	Pericardium	-	Solitary cardiac	Yes	-	[24]
2012	Markovic et al.	Myxoid liposarcoma (thigh)	45	Pericardium	5 years	Solitary cardiac	Yes	Patient still alive at 6-month follow-up	[25]
2011	Mitomi et al.	Dedifferentiated liposarcoma (retroperitoneal)	71	LV, pericardium	1 year	Lungs	No (expired soon after diagnosis)	Confirmed death 2 days later	[26]
2011	Dogan et al.	Myxoid liposarcoma (thigh)	54	LA, LUPV	4 years	Solitary cardiac	Yes	Confirmed death 10 months later	[27]
2011	Lazopoulos et al.	Myxoid liposarcoma (thigh)	63	LV, pericardium	13 years	Abdomen	Yes	Patient disease-free at 6-month follow-up	[28]
2011	Ribeiro et al.	Myxoid liposarcoma	70	LV	At initial diagnosis	Abdomen, lungs, pleura	No (extensive disease)	Palliative care only	[29]
2009	Komoda et al.	Myxoid liposarcoma (thigh)	52	RA, RV, epicardium, AV sulcus	17 years	Retroperitoneum	Yes	Patient still alive at 30-month follow-up	[30]
2007	Chughtai et al.	Pleomorphic liposarcoma (shoulder)	46	RV	3 years	Lungs	Yes	-	[31]
2005	Fairman et al.	Myxoid liposarcoma (thigh) (LG)	56	LV	12 years	Solitary cardiac	Yes	-	[32]
2005	Kono et al.	Myxoid liposarcoma (lower limb)	60	RA, RV, SVC	13 years	Solitary cardiac	Yes	Patient still alive at 10-month follow-up	[33]
2005	Aoyama et al.	Myxoid liposarcoma (thigh)	63	Pericardium	1 year	Pleura	Yes	Confirmed death soon after the operation	[34]
2002	Lee et al.	Myxoid liposarcoma (thigh)	53	Extensive cardiac	5 years	Solitary cardiac	Yes	Confirmed death during the operation	[35]
2002	Wong et al.	Myxoid liposarcoma (chest)	54	RV	7 years	Solitary cardiac	Yes	Patient had stable disease at 6-month follow-up	[36]
2001	Ng et al.	Myxoid liposarcoma (thigh)	45	Interventricular septum, paracardiac region	3 years	Thighs, mediastinum, iliac nodes, adrenal, azygo-oesophageal space	No (extensive disease)	Non-cardiac disease progression 11-month follow-up	[37]
2000	Sugiyama et al.	Myxoid liposarcoma	61	RV	11 years	Solitary cardiac	Yes	Confirmed death 6 days after the operation	[38]
2000	Gacem et al.	Myxoid liposarcoma (groin)	57	Pericardium	13 years	Solitary cardiac	Yes	Patient still alive at 36-month follow-up	[39]
1997	Hatton et al.	Myxoid liposarcoma	42	Pericardium	3 years	Abdomen, pelvis, lungs	No (extensive disease)	Confirmed death 1 year later	[40]
1997	Wilhelmi et al.	Myxoid liposarcoma (LG)	53	Pericardium	9 years	Solitary cardiac	Yes	Patient still alive at 7-month follow-up	[41]
1994	Papa et al.	Myxoid liposarcoma (thigh)	45	LV	15 years	Abdomen	No (tumor deemed as nonresectable)	Patient still alive at 6-month follow-up	[42]
1993	Oshima et al.	Myxoid liposarcoma (thigh)	37	LV	5 years	-	-	-	[43]
1992	Langlard et al.	Myxoid liposarcoma (thigh)	54	RV	5 years	Solitary cardiac	Yes	Confirmed death 2 years later	[44]
1991	Maloisel et al.	Myxoid liposarcoma (lower limb)	28	RA	4 years	Lungs	No	Confirmed death 3 months later	[45]
1990	Schrem et al.	Myxoid liposarcoma (knee)	41	RA	5 years	Solitary cardiac	Yes	Patient still alive and in good condition at 1-year follow-up	[46]
1988	Ozoux et al.	Myxoid liposarcoma (thigh)	60	LV	17 years	Retroperitoneum	Yes	Confirmed death 6 months later	[47]
1988	Bartels et al.	Liposarcoma	64	RV, PA	17 years	Solitary cardiac (at diagnosis)	Yes	Confirmed death 14 hours after the operation	[48]
1986	Lagrange et al.	Myxoid liposarcoma (thigh)	39	RV	6 years	Solitary cardiac	Yes	Patient disease-free at 10-month follow-up	[49]
1985	Pizzarello et al.	Liposarcoma	61	RA, RV, pericardium	20 years	Pleura	No (Death soon post-admission)	Confirmed death 6 days later	[50]
1983	Ravikumar et al.	Myxoid liposarcoma (thigh)	76	Pericardium, anterior wall	25 years	Solitary cardiac	Yes	Patient disease-free at 1-year follow-up	[51]
1981	Godwin et al.	Myxoid liposarcoma (thigh)	59	RV, Pericardium	25 years	Solitary cardiac	Yes	-	[52]
1981	Mavroudis et al.	Myxoid liposarcoma (thigh)	59	RV, Pericardium	25 years	Chest, diaphragm, retroperitoneum	Yes	Patient disease-free at 7-month follow-up	[53]
1968	Tong et al.	Myxoid liposarcoma (thigh)	35	LV	7 years	Groin	No	Patient still alive at 30 months follow-up	[54]
1939	Scott et al.	Liposarcoma	-	Heart, pericardium	-	-	-	-	[55]

**Table 1:** Published cases of myxoid liposarcoma metastatic to the heart. We identified 46, other than ours, reported cases of liposarcoma metastatic to the heart. In 36 of the cases (77%), the metastases originated from soft tissue sarcomas, in 4 (9%) from visceral, and in 7 of them (15%), the location of the primary tumor was not specified. Out of the soft tissue sarcomas, 34 (94%) of the 36 originated from the lower limbs. Regarding the histopathology, 40 (85%) of them were classified as myxoid (or round cell) liposarcomas, 3 (6%) as dedifferentiated, 1 (2%) as pleomorphic, and 3 (6%) remained unspecified by the authors. Tumor grade information, when provided, is noted in the table as either low grade (LG) or high grade (HG). The average age of diagnosis of cardiac metastasis was 52.5 years, and on average occurred 8.5 years following the diagnosis of the primary tumor. Twenty-seven of the metastases (57%) involved only one cardiac location, while 20 of them (43%) involved more than one location. The pericardium was the location most commonly involved (16 cases) (34%), followed by the left ventricle (LV) (15 cases) (32%), the right ventricle (RV) (14 cases) (30%), and the right atrium (RA) (9 cases) (19%). Surprisingly, the left atrium (LA) was involved only in 1 of the 47 reported cases (2%). Out of the 45 cases describing the metastatic burden of the patients at the time of diagnosis, 18 (40%) of them had at least one additional extracardiac metastatic locus, while 27 (60%) of them had solitary cardiac metastasis. Surgery was the preferred method of management in 30 (67%) of the 45 cases discussing management. Twenty-four of them (80%) happened in patients with solitary cardiac lesions, and the balance in patients with multifocal metastatic disease. Particularly, other than our patient, the other two patients with solitary cardiac lesions who did not receive cardiac surgery expired before a therapeutic plan was implemented. Out of the 38 cases detailing the patient’s outcome, 19 (50%) reported patient deaths before the manuscript’s publication, predominantly within the initial year post-diagnosis of the cardiac metastasis. The remaining 50% indicated that patients were alive at the time of article publication, with the longest reported survival time, aside from our patient’s, being 3 years.

## Data Availability

Further information can be provided upon request to the corresponding author of the manuscript.
